# Experiences of cohabiting partners of women diagnosed with cancer during pregnancy: a qualitative study

**DOI:** 10.1007/s00520-024-08570-8

**Published:** 2024-05-27

**Authors:** Michelle Sinclair, Richard Song, Michelle Peate, Christobel Saunders, Jocelyn Lippey, Mark P. Umstad, Kylie Mason, Angela Ives, Lesley Stafford

**Affiliations:** 1 Department of Surgery, The University of Melbourne, Parkville, VIC Australia; 2 Department of Rural Health, Melbourne Medical School, The University of Melbourne, Shepparton, VIC Australia; 3 Department of Obstetrics and Gynaecology, Royal Women’s Hospital, Parkville, VIC Australia; 4 The University of Melbourne, Parkville, VIC Australia; 5 Medical School, The University of Western Australia, Perth, WA Australia; 6 Department of Surgery, St. Vincent’s Hospital, Fitzroy, VIC Australia; 7 Department of Obstetrics and Gynaecology, The University of Melbourne, Parkville, VIC Australia; 8 Department of Maternal-Fetal Medicine, The Royal Women’s Hospital, Parkville, VIC Australia; 9 Department of Clinical Haematology, Peter MacCallum Cancer Centre, Royal Melbourne Hospital, Parkville, VIC Australia; 10 Department of Oncology, Sir Peter MacCallum, University of Melbourne, Parkville, VIC Australia; 11 Familial Cancer Centre, Royal Melbourne Hospital, Parkville, VIC Australia; 12 Melbourne School of Psychological Sciences, University of Melbourne, Parkville, VIC Australia

**Keywords:** Cancer, Gestational, Spouses, Caregivers, Psychosocial support systems, Pregnancy, Psycho-oncology

## Abstract

**Purpose:**

When a pregnant woman is diagnosed with cancer, she faces complex and unique challenges while navigating both obstetric and oncological care. Despite often being the primary support for women diagnosed with cancer during pregnancy (CDP), little is known about the experiences of their partners. We undertook an in-depth exploration of the experiences of partners of women diagnosed with CDP in Australia.

**Methods:**

Semi-structured interviews were conducted with partners of women diagnosed with CDP treated in Australia. Interviews explored partners’ inclusion in decision making and communication with health professionals and their own coping experiences. Data were analysed thematically.

**Results:**

Data from interviews with 12 male partners (*N* = 12) of women diagnosed with CDP were analysed. Two unique themes relevant to partners were identified: ‘Partners require support to adjust to changing roles and additional burdens’ and ‘Treating the couple as a team facilitates agency and coping, but partners’ needs are placed second by all’.

**Conclusion:**

Partners of women diagnosed with CDP commonly experience unique stressors and a substantial shift in previously established roles across multiple domains including medical advocacy, household coordination and parenting. Partners’ coping is interlinked with how the woman diagnosed with CDP is coping. Inclusion of partners in treatment decisions and communications, and considering partners’ wellbeing alongside that of the woman with CDP, is likely to be supportive for partners. In turn, this is likely to enhance the quality of support that women diagnosed with CDP receive from their partners.

**Supplementary Information:**

The online version contains supplementary material available at 10.1007/s00520-024-08570-8.

## Introduction

When a woman is diagnosed with cancer during pregnancy (CDP), she is faced with a potentially life-limiting illness, the implications of which may have far-reaching consequences for her and her family. Optimal oncologic treatment for the woman may compromise the pregnancy [1]. Treating CDP therefore requires a multidisciplinary approach to balancing oncologic outcomes for the woman, while mitigating risks to the foetus presented by diagnostic tests and oncologic treatments [2].

Clinical practice guidelines help navigate the complexities of treatment for CDP and detail the current best practice for the type, stage and location (where applicable) of cancer and the gestation of the foetus [3, 4]. While pregnancy itself is not a predisposing factor for developing cancer or necessarily indicative of a poorer prognosis, misattribution of cancer symptoms as pregnancy-associated symptoms can contribute to delayed diagnosis and result in a more advanced stage of disease at diagnosis [5]. Some diagnostic tests and treatments are considered safe during all stages of pregnancy (such as ultrasound and some surgeries); others are contra-indicated during certain critical periods of foetal development, particularly the first trimester (such as some chemotherapy regimens), and some are only recommended after the birth (such as radiotherapy) [3]. For some presentations, delaying treatment is not recommended, and protection of the foetus during treatment is not possible. In these cases, in consultation with the family, therapeutic abortion may be recommended [6].

It is crucial that the complexities of treatment planning are considered within the context of the patient’s needs and preferences [2]. At a time often spent excitedly preparing for a newborn, women diagnosed with CDP and their partners may therefore instead face difficult, time-sensitive treatment decisions that have implications for the woman’s and the foetus’ wellbeing. Our understanding of the needs of women diagnosed with CDP is growing [7, 8]; however, little is known about the experiences of their partners. Partners comprise an important population in their own right, but are also commonly the key providers of support for women diagnosed with cancer and in many instances, the co-parent of the pregnancy affected by cancer.

The importance of partners in supporting women who have cancer but are not pregnant is well-known. The extent to which a mother perceives her partner to be supportive has been associated with lower maternal and infant distress and better quality of life [9, 10]. Similarly, in the oncology context, partners play a significant role in supporting the wellbeing of cancer patients and experience similar rates of psychological distress to the index patient population [11–14]. Distress in partners may also be associated with increased distress in the patient [15, 16]. Indeed, it has been shown that a cancer diagnosis is likely to impact a couple as a dyad, with both the partners’ and the cancer patients’ coping styles exerting mutual influence over one another [17].

Unsurprisingly, therefore, extant literature suggests that partner support is also essential for the wellbeing of the woman diagnosed with CDP. Support from partners was one of three themes identified by Faccio and colleagues in their qualitative analysis of motherhood during or after breast cancer [18]. Similarly, in a qualitative analysis of the healthcare experiences of women diagnosed with breast cancer either while pregnant or within 12 months post-partum, women reported that they predominantly relied on their partners to manage the distress of the diagnosis [19]. However, only two studies have included the perspectives of partners of women diagnosed with CDP. Vandenbroucke and colleagues [20] assessed the coping styles of 61 couples affected by CDP. The authors found that women diagnosed with CDP and their partners’ levels and patterns of distress and coping were interrelated; however, partner coping was not explored in depth. Gomes et al. [21] interviewed 12 women diagnosed with CDP and 19 family members, including nine partners; however, families were interviewed together and the experiences of partners were not analysed or reported separately. This is important as at the time of data collection, partners may have restricted their responses to minimise potential distress in the woman with cancer.

As partners and patients may experience similar levels and patterns of distress and coping, and women diagnosed with CDP face multifaceted challenges, it is important to understand the experiences and needs of the partners of these women independently. Thus, this study aimed to explore the experiences of partners of women diagnosed with CDP in Australia with a focus on supportive care needs.

## Methods

The INTEGRATE (Experiences of Pregnant Women with Cancer: Exploring Parenting and Mental Health Needs) study explored the experiences and needs of (a) women diagnosed with CDP, (b) their partners and (c) the clinicians who treat this population. Women diagnosed with cancer post-partum or diagnosed with gestational trophoblastic disease were not eligible. Findings from INTEGRATE relating to the healthcare and psychosocial experiences of women diagnosed with CDP and the experiences of health professionals treating this population have been previously published [22–24]. Here we report on the experiences of partners of women diagnosed with CDP.

INTEGRATE received ethics approval from the Royal Women’s Hospital Human Research Ethics Committee in 2018 (ID#18/25).

### Recruitment

National recruitment occurred over 6 months from 2018 to 2019. Participants were recruited through targeted advertisements distributed through professional, clinical and community networks and via social media, or through women already participating in INTEGRATE. Advertisements directed potential participants to a website that hosted study details including eligibility and consent information and invited potential participants to express their interest in participating. These potential participants were contacted by a member of the study team, given the opportunity to ask questions, and after confirmation of eligibility, a telephone interview was scheduled.

### Eligibility

A partner was defined as a designated support person of a woman diagnosed with CDP who (a) had the potential to be involved in treatment decisions, appointments and clinical communication and (b) provided practical and emotional support considered instrumental to CDP care. Consequently, they did not need to be a co-parent of the pregnancy or in an intimate relationship with the woman diagnosed with CDP.

Partners were eligible if the woman they supported was diagnosed with any CDP in the previous 5 years, and if the woman diagnosed with CDP had received treatment within Australia. Participants were also required to be at least 18 years old, able to provide informed consent and have sufficient English language proficiency to participate in the interview. The pregnancy outcome (e.g. live birth/termination of pregnancy/foetal death in utero) did not affect eligibility. As per eligibility for INTEGRATE, partners of women diagnosed with cancer after pregnancy or diagnosed with gestational trophoblastic disease were not eligible for participation.

### Data collection

The interview schedule was developed by a multidisciplinary team representing psycho-oncology, clinical psychology, perinatal psychiatry, medical and surgical oncology, haematology, obstetrics and two consumers with previous diagnoses of CDP. Interview questions were chosen following a literature search (e.g. [8, 19, 20]) and based on areas deemed relevant to the healthcare experiences and psychosocial needs of partners as determined by the clinical expertise of the multidisciplinary team. Questions explored the partners’ perspectives, experiences and needs associated with the cancer diagnosis, treatment decisions and appointments, interactions with health professionals and impact on family life (see Appendix for interview schedule).

Telephone interviews were conducted by two psychologists with research interview experience and were audio recorded. Prior to the recording commencing, socio-demographic data including gender, age, number of children (if any), relationship to the woman diagnosed with CDP, highest level of education and place of residence were collected. Clinical data about the woman diagnosed with CDP including details of the cancer diagnosis and treatment and, where appropriate, the gestational age at which the cancer was diagnosed and pregnancy outcome were also collected.

### Analysis

Interview recordings were transcribed verbatim, entered into NVivo 12 software and then examined using thematic analysis as per the Braun and Clarke method [25]. The analysis followed the quality assurance criteria outlined by Nowell and colleagues to enhance reliability and the trustworthiness of the data [26]. The interviewers frequently shared field notes and, prior to formal coding, identified initial impressions and codes. One fifth of the interviews were independently coded by two researchers to identify further tentative codes. All codes were then discussed with the lead investigator until a consensus on preliminary codes was reached. These codes were applied to the remaining transcripts and grouped into potential themes which were revisited against lower order codes and the original dataset. It became apparent that several themes resembled data previously reported in women diagnosed with CDP [19, 23]. Consequently, themes unique to partners became the focus. Thematic meaning and content were established and potential sub-themes were identified. Once no new themes unique to partners’ experiences were identified within the collected data, it was determined that the data had been sufficiently explored and that data saturation had been reached [27]. Illustrative quotes were then selected.

## Results

### Sample

Sixteen partners of women diagnosed with CDP consented to participate, with two lost to follow-up (one had no availability during the scheduled interview times; the other could not be contacted). The interviews of two female non-cohabiting partners (one sibling and one parent) revealed inherently different experiences with cohabiting partners. Subsequently, the data from these non-cohabiting partner interviews were excluded from the current analyses, and data from cohabiting partners became the focus. Henceforth, the term ‘partner’ is used to indicate ‘cohabiting partner’.

The final sample consisted of 12 interviews all of whom were male cohabiting partners of women diagnosed with CDP. Interviews lasted an average of 52 min (SD = 16.7, range = 31–79), and the average age of participants at the interview was 36.8 years (SD = 3.2, range = 33–41). Most participants were the partners of women who had been diagnosed with breast cancer (*n* = 8, 66.7%) or who had had a live birth (*n* = 8, 66.7%). See Table 1 for the socio-demographic characteristics of the partner sample and information about the women diagnosed with CDP.

**Table 1:** Characteristics of participants and women diagnosed with CDP

**Partner characteristics**	
	**N (%)**
Male	12 (100.0%)
No prior children at time of CDP	4 (33.3%)
**Highest level of education**	**N (%)**
Undergraduate degree	7 (58.3%)
Post-graduate degree	2 (16.7%)
Trade/TAFE/Certificate	3 (25.0%)
**State/province**	**N (%)**
Victoria	5 (41.7%)
New South Wales	5 (41.7%)
Northern Territory	1 (8.3%)
Western Australia	1 (8.3%)
**Details of women diagnosed with CDP**	
**Diagnosis**	**M (SD) range**
Gestation at diagnosis (weeks)	13.8 (6.0) 5.0–23.0
Time since diagnosis (months)	24.4 (23.4) 1.0–72.0
**Outcome/status of pregnancy at time of interview**	**N (%)**
Live birth	8 (66.7%)
Currently pregnant	3 (25.0%)
Termination	1 (8.3%)
**Cancer type**	**N (%)**
Haematology	2 (16.7%)
Breast	8 (66.7%)
Cervical	1 (8.3%)
Lung	1 (8.3%)

*CDP* cancer during pregnancy

### Themes

Some of the experiences of partners echoed those reported by women diagnosed with CDP [19, 23]. These included that:

- Tailored and well-coordinated care which addressed the unique presentation of CDP was perceived as supportive [19, 23]
- Trust in clinicians and service providers, which was influenced by the quality and style of communication, and the perception of competence of the treating staff and facility, significantly contributed to a positive healthcare experience [23]
- There is a strong need to promptly address the psychosocial and parenting needs of women diagnosed with CDP and their partners [19, 23]

To focus on novel findings, here we report on the themes unique to partners. Two interrelated themes were identified: ‘Partners require support to adjust to changing roles and additional burdens’ and ‘Treating the couple as a team facilitates agency and coping, but partners’ needs are placed second by all’.

#### Theme 1: Partners require support to adjust to changing roles and additional burdens

Partners were very aware of the unique challenges faced by the woman diagnosed with CDP. They sought to protect her wellbeing through the minimisation of treatment burden and household coordination. For the former, this involved adopting new roles including medical advocacy, liaison and appointment coordination. For the latter, previously established roles within the partnership/family shifted and partners accepted increased parenting, financial and household coordination responsibilities. All partners described a significant adjustment period following the CDP diagnosis characterised by needing to adapt to the added load of new responsibilities while simultaneously meeting the practical challenges posed by the diagnosis, cancer treatment and treatment-related side effects.

It’s stressful at times… I’ve had to take on the majority of the parenting, the majority of the household duties in order for [name of woman diagnosed with CDP] to stay in a positive manner mentally, so it’s difficult. P040, 36 years old.

Parenting was a key area where partners increased their involvement as the women with CDP had reduced availability to parent due to medical appointments and treatment side-effects (e.g. fatigue, inability to breastfeed due to treatment toxicity). Most partners reported that this resulted in a deeper connection with their children and/or the infant of the CDP pregnancy. Partners were also cognisant of distress associated with reduced availability to parent experienced by the woman diagnosed with CDP and attempted to protect mother–child time. For example, most partners were included in discussions about breastfeeding and where breastfeeding was disrupted or not possible; some reflected on the disappointment experienced by the woman diagnosed with CDP. However, the partners themselves often expressed gratitude that bottle-feeding enabled them to be more ‘hands-on’ during this time and that this involvement facilitated stronger bonding with their child.

I was… thrust into fatherhood fairly quickly due to the fact that [she] had surgery and went back into hospital again, so I was playing fulltime dad at one stage. [She] was in hospital for a week and a half, so I had a newborn at home. She couldn’t pick him up or anything like that… so I was on duty at night time for feeds 100% of the time. Yes, it was a workload, but at the end of the day [she] needed the rest, and the help, and I was there to do that. P031, 34 years old.

[She] had seven weeks radiation... So, I had a really good connection [with name of the child born from the pregnancy affected by cancer] …and still do today just because I’ve had that role from day one. P059, 40 years old.

Financial concerns were a challenge for partners. Expenses arising from treatment and a newborn were compounded by the woman diagnosed with CDP not working and the partner’s reduced capacity to work. These financial stressors typically detracted from partners’ wellbeing. For some partners, though, work provided distraction and routine that supported their coping.

My immediate concern was how…was I going to pay for it all... This is going to be a really really… costly and expensive journey…we have got a baby on the way, [woman diagnosed with CDP is] going to be out of work, I’m going to be out of work. That was my initial thought…how… are we going to pay for this? P069, 33 years old.

Some partners expressed a lack of confidence in the care that the woman diagnosed with CDP was receiving, resulting in the perception that medical advocacy was an additional role they had to assume. Partners expressed the view that the healthcare system is not set up to treat women diagnosed with CDP and that without the partners’ active and vigilant advocacy, important aspects of care could be missed. In these instances, the partner took on the responsibility of either finding medical care in which they had more confidence, or questioning treatment changes and plans.

But that’s what frustrated me if I hadn’t have jumped up and down and carried on like a… pork chop then we would have been on a treatment path that probably wouldn’t have been in [the woman diagnosed with CDP’s] best interest… we wouldn’t have been getting the best care...by the people that…should have been looking after her and see this stuff on a regular basis. P069, 33 years old.

When it was clear to partners who was responsible for which aspect of the woman’s care (there was a clear delineation of responsibilities) and partners felt they could trust the medical aspects of this care, they reported better coping and improved capacity to support the emotional wellbeing of the woman diagnosed with CDP.

So he [the doctor] said ‘Well my job is to do all of the haematology and I can do that. But your job is to hold everything else together and I can’t do my job unless you do that’… we had an understanding. P017, 41 years old.

#### Theme 2: Treating the couple as a team facilitates agency and coping, but partners’ needs are placed second by all

Partners found it supportive of their wellbeing when they were included in communication and treatment decisions by clinicians, that is, when the partner and the woman were treated as a team by the healthcare team. Such inclusion translated into perceptions of enhanced efficacy to problem solve and manage pragmatic tasks.

I had a few questions as well about [the woman with CDP], so it was nice to be able to ask the questions directly and get some answers back… half the time my frustration is being left out of the loop…I’m trying to organise home in the background, I… want to be included in the all the conversations so I know what I have to plan for next. P040, 36 years old.

When they were not included in treatment discussions or decisions, partners believed this had a negative impact on the woman diagnosed with CDP, sometimes to the extent that her care could be compromised. This responsibility weighed heavily on some partners, as demonstrated here where the woman with cancer was very unwell and her partner was not included in communications regarding a change in treatment plan:

I’ve gone to this guy who’s then changing all of her treatment and I can’t talk to him, no-one’s explaining to me what's going on. And so then I just demanded that they continue the treatment that she was already on. And they did it… I can’t be saying: Oh well, I should have done that… I have to be happy that what I’ve done… if [the woman with CDP] dies. P017, 41 years old.

Partners prioritised the wellbeing of the woman diagnosed with CDP and placed their own needs second. The extent to which partners acknowledged their own distress varied among participants. Some were more inclined to minimise their experiences and focus on those of the woman diagnosed with CDP, while others described noticeable psychosocial and physical manifestations of their distress. Where available, partners relied on pre-existing support networks for practical and emotional support.

She was the priority… It was… her that was going through all this stress but there’s stress that I was going through as well… that pales in comparison to what she was going through. So… that doesn’t matter at the end of the day. P018, 40 years old.

[I was] pretty stressed out. I’ve been getting chest pains and high blood pressure… which never presented itself before this. P069, 33 years old.

Your attention’s on the wife or partner… it’s quite an overwhelming experience. If you didn’t have such good family support like we had, it could’ve been a big challenge. A bigger challenge I should say. P061, 35 years old.

Partners appreciated offers of formal (such as seeing a psychologist) and informal (acknowledgements of their coping from health professionals) support. Barriers to accessing support included the limited capacity to attend or make appointments (too busy to call and organise), inflexible appointment times or setting offered (required in-person attendance) and struggling with treatment burden and the quantity of appointments required. Many of these barriers were exacerbated when partners placed the woman diagnosed with CDP’s needs above their own.

It was so rare that we’d actually have a day without anything... when those days came around we didn’t want yet another appointment or another phone call… so we… ended up sacrificing those mental health days for just a day off… And to have me out of the house for an hour or two… was just too difficult. P087, 33 years old.

## Discussion

Partners of women diagnosed with CDP are an important but neglected population. This is the first study to provide an in-depth exploration of the needs specific to this group. Previously, it has been found that trust in the treating team, effective communication and coordination of care are central to a positive healthcare experience for pregnant women with cancer [19, 23]. Partners in our sample expressed many of these same sentiments. Unique to partners were the challenges they faced in adjusting to new roles and increased demands following the diagnosis of CDP, the direct negative impact on them when excluded from important treatment decisions and communications and their focus on the woman diagnosed with CDP’s coping over their own.

It is well established that partners of cancer patients can find the adjustment to an increased support role anxiety-provoking [13]. For our sample, the added responsibilities in the areas of parenting, finances and medical liaison were significant stressors. Of particular importance was the finding that when partners were not confident in the medical care, they took on the role of medical advocacy to the extent that they believed that ensuring adequate care was their responsibility. This further underpins the need for experienced clinicians to be involved in the care of women diagnosed with CDP [22], and for clear, comprehensive communication with patients and their partners to be prioritised [19, 23]. Both are associated with greater trust in treating clinicians in women diagnosed with CDP. Partners would likely benefit from reassuring and transparent communication regarding the competence and experience of treating clinicians, medical care and treatment planning. Where appropriate, the direct involvement of partners in the treatment decisions, communications and care coordination, alongside women diagnosed with CDP would likely also be supportive. Cancer-specific clinical practice guidelines also provide current best practice procedures and treatments for women diagnosed with different cancers (e.g. gynaecological cancer [28], haematological [29], breast [30], lung [31]). Transparent communications with families regarding consultation of these guidelines may bolster confidence in clinicians.

A recent review identifying ethical concepts covered in the treatment guidelines for CDP found patient autonomy dominated [32]. While relational autonomy—the involvement of the partner and or family in decision-making—was included within this principle, reference to relational autonomy was only found in 10 of the 25 included guidelines. There is a need for more explicit direction from disciplinary experts regarding the benefits of the inclusion of partners and support in decision-making conversations. We found inclusion in care was pivotal for partners’ coping. Inclusion provided the scaffolding on which further coping was built. In our sample, this meant that when partners were included in treatment and care discussions by clinicians and felt supported, they reported more capacity to support the woman diagnosed with CDP and in turn, their own coping improved. Our findings further reinforce the importance of holistic and comprehensive multi-interdisciplinary care for families affected by cancer diagnosed during pregnancy. Patient-centred interdisciplinary care involving collaboration between oncologic, obstetric, neonatal, psychosocial, pharmacological and administrative teams is key to adequately supporting these patients and their families [2].

The need for increased and accessible support for partners of women diagnosed with CDP has been previously identified [23]. While partners acknowledged the challenges they faced and that their inclusion in treatment decisions and communication was beneficial, they also reported a preference for available support to be directed to the woman diagnosed with CDP and reduced capacity to engage with needed support. It may be that partners can acknowledge that they have a need for support, but that it is difficult to accept or prioritise meeting these needs if they perceive the needs of the woman with CDP to exceed their own. Partners may benefit from an acknowledgement of their difficult position and encouragement to access additional support for themselves. Practically, this may take the form of clinician-initiated referrals for psychosocial support and targeted discussions regarding partner and family wellbeing. Health professionals could prioritise offers of accessible and flexible support options such as online written or video resources or telehealth to facilitate partner engagement.

## Limitations

Here we provide the first in-depth investigation of the experiences of partners of patients diagnosed with CDP. While partners of women diagnosed with any cancer during pregnancy and any pregnancy outcome were eligible, most participants were partners of women diagnosed with breast cancer and who had experienced a live birth. We purposefully did not include women diagnosed with cancer in the post-partum. The generalisability of these findings to partners of women diagnosed with other cancers, with other pregnancy outcomes or diagnosed in the post-partum is limited. Data on ethnicity were not collected and participants who could not participate in English were not eligible, both of which are limitations. We also acknowledge that while CDP is rare, the sample size is small and limited in educational diversity which may impact the generalisability of the findings.

## Future research

While it has previously been suggested that the diagnosis of CDP may interrupt mental representations of the future child and potentially disrupt maternal attachment [18], the impact on the partner’s bond has not been explored and could be an important area for future research. Our findings suggest that for some partners, taking on additional parenting increased opportunities for deeper bonding with their children post-pregnancy. Future research could also explore whether and how the division of parental responsibilities shifts after the intensity of cancer treatment ceases. Future revisions and updates to clinical practice guidelines for treating CDP should contain guidance on the inclusion of partners and support persons in important treatment planning discussions.

## Conclusion

To our knowledge, this is the first study to provide a detailed exploration of the unique experiences of partners of women diagnosed with CDP. These partners must adjust to the significant upheaval of previously established life roles with the added complexities around adjusting to a newborn, taking on a larger proportion of parenting than previously planned, providing increased practical, emotional and financial support for their family while simultaneously attempting to facilitate the coordination of appointments across two healthcare systems. Clinicians can support these families by including partners in care decisions and communications and prioritising the wellbeing of partners alongside their patients. It is important to understand that partner and patient coping are interrelated: When partners’ coping and wellbeing are supported by clinicians, they are likely to have an increased capacity to provide better support for the women diagnosed with CDP.

### Supplementary Information

Below is the link to the electronic supplementary material.

Supplementary file1 (DOCX 23 KB)

## Data Availability

The datasets generated during and/or analysed during the current study are available from the corresponding author on reasonable request.
