# Supplementary material for: Experiences of cohabiting partners of women diagnosed with cancer during pregnancy: a qualitative study
Source: Support Care Cancer. 2024 May 27;32(6):384. doi: 10.1007/s00520-024-08570-8 (PMC11129963; doi:10.1007/s00520-024-08570-8)
Supplement: Supplementary file 1 — Supplementary file1 (DOCX 23 KB) [file 520_2024_8570_MOESM1_ESM.docx]

Appendix- Interview schedule

| Diagnosis | - Could you talk about the experience leading up to your partner’s diagnosis? |
| --- | --- |
| Interdisciplinary care | - Can you tell me about how you decided on where your partner received her maternity and oncology care? - Could you tell me a bit about what was it like for you and your partner to be treated across two different types of medical teams, and potentially across sites and systems? |
| The baby | - What were your immediate concerns for the baby/pregnancy and your partner? - What were your thoughts/ feelings towards the baby? - Have there been differences between what you wanted and what your partner wanted in relation to continuing the pregnancy? - How did you manage this? - Were you satisfied with how information was communicated to you about the baby? - Who (which clinician) communicated with you about this? - Was there any discussion with you about potential risks to the unborn baby? - Was there any discussion with you about the baby’s long-term development after birth? - Did your partner consider ending her pregnancy? - Was she advised to do so? - What were your thoughts? |
| Communication across treatment | - What was your general experience of communication with the treating teams? - To what extent were you included in these conversations? - What could have been improved in relation to communicating with you about your partner’s health/baby’s health? |
| fertility | - Was your partner’s future fertility discussed? - Were you included in this conversation? - Were you satisfied with the information you received? |
| treatment and side effects | - How much were you involved in treatment decisions? - What was your partner’s experience of treatment and side effects? - How did side-effects impact on your family? |
| Wellbeing | - Were you asked about how you were feeling or coping with your partner’s diagnosis/treatment? - Were you satisfied with how your concerns were addressed? - Was there anything else clinicians could have done to be more supportive? - How do you feel now about your partner’s diagnosis? - How do you feel now about the pregnancy/loss of pregnancy? - Was there recognition of your role as the partner/ father of the pregnancy? |
| Birth | - Have you had discussion about delivery? - How have they been managed? - What information about the delivery did/do you want? - Are/ were you satisfied with the information you received? |
| Breast feeding | - Has breastfeeding and bottle feeding been discussed with you? - Have you been included in these conversations? - Were you satisfied with these discussions? |
| After the birth | - Did your partner have further treatment after the baby was born/ is your partner expected to have further treatment after the baby is born? - How did this affect your family? - How did this affect your parenting? - How did your partner manage separation from the baby? - How did this affect your relationship with your partner? - Positive and negative changes, overall? - Are there things that would have helped you after the miscarriage/termination/birth? |
| parenting | - Thinking about the baby from the gestational cancer pregnancy: Has your partner’s cancer diagnosis affected how you interact with your child? - What are your thoughts/feelings towards this child? - How has this experience affected your thoughts about the sort of parent you want to be for your child? - Do you have any anxieties about how your experiences have affected your emotions and feelings towards you child? - What do you most want your child to know about you as a parent? - Do you think your child is affected by your moods and emotional state? - How do you work out what your child needs from you? - What ideas do you have about your child’s emotional needs? - What might help you in helping your child feel secure? - Thinking about other children that you may have or your wider family: How has your partner’s cancer diagnosis and treatment affected your family/ other children? - What are your thoughts/feelings towards other children? |
| Model of care | - In your experience: How well does the current model of care work i.e., where you can receive care at different sites, potentially across public and private health? |
|  | - Is there anything else you would like to add that you think we should know in developing support for women, families, partners, clinicians and how we structure ca |
